# POU2F1 over-expression correlates with poor prognoses and promotes cell growth and epithelial-to-mesenchymal transition in hepatocellular carcinoma

**DOI:** 10.18632/oncotarget.17296

**Published:** 2017-04-20

**Authors:** Yonghao Zhong, Hongyang Huang, Min Chen, Jinzhou Huang, Qingxia Wu, Guang-Rong Yan, De Chen

**Affiliations:** ^1^ Biomedicine Research Center, The Third Affiliated Hospital of Guangzhou Medical University, Guangzhou, China; ^2^ Department of Surgery, The Third Affiliated Hospital of Guangzhou Medical University, Guangzhou, China; ^3^ Key Laboratory of Protein Modification and Degradation, Guangzhou Medical University, Guangzhou, China

**Keywords:** POU2F1, hepatocellular carcinoma

## Abstract

Despite recent efforts to understand activities of POU domain class 2 transcription factor 1 (POU2F1), little is known about the roles of POU2F1 in hepatocellular carcinoma (HCC) tumorigenesis and its correlation with any clinicopathological feature of HCC. In this study, we found that POU2F1 was significantly up-regulated in HCC specimens compared with adjacent non-cancerous liver specimens. The high POU2F1 protein expression level positively correlated with large tumor size, high histological grade, tumor metastasis and advanced clinical stage, and HCC patients with high POU2F1 levels exhibited poor prognoses. We further demonstrated that POU2F1 over-expression promoted HCC cell proliferation, colony formation, epithelial-to-mesenchymal transition (EMT), migration and invasion, while silencing of POU2F1 inhibited these malignant phenotypes. *POU2F1* induced the expression of *Twist1*, *Snai1*, *Snai2* and *ZEB1* genes which are involved in the regulation of EMT. Furthermore, POU2F1 was up-regulated by AKT pathway in HCC, and POU2F1 over-expression reversed the inhibition of malignant phenotypes induced by AKT knock-down, indicating POU2F1 is a key down-stream effector of AKT pathway. Collectively, our results indicate that POU2F1 over-expression is positively associated with aggressive phenotypes and poor survival in patients with HCC, and POU2F1 regulated by AKT pathway promotes HCC aggressive phenotypes by regulating the transcription of EMT genes. POU2F1 may be employed as a new prognostic factor and therapeutic target for HCC.

## INTRODUCTION

Hepatocellular carcinoma (HCC), the sixth most prevalent type of cancer, is the fourth leading cause of cancer-related morbidity and mortality in China [1, 2]. The prognosis of HCC patients remains poor, mainly because the recurrence rates are high even after surgical resection; tumor recurrence complicates more than 70% of cases at five years after resection [3, 4]. Although surgical resection is a potentially curative treatment for HCC, clinical outcomes of HCC are still unfavorable [5]. Therefore, it is necessary to find novel therapeutic targets to improve the prognosis of patients with HCC.

POU2F1 is located on chromosome 1q24, which is also known as octamer binding transcription factor-1 (OCT-1) [6]. It is a ubiquitous transcription factor that regulates the transcription of target genes associated with cell cycles [7]. POU2F1 is involved in the cell differentiation through regulation of housekeeping genes like H2B and snRNAs, and it also participates in immunity and inflammation via modulation of tissue-specific target gene expression [8, 9]. Additionally, POU2F1 was shown to participate in cellular response to DNA damage [10, 11]. It was reported to be over-expressed in osteosarcoma tumors and identified as an independent prognostic factor in gastric carcinoma [12, 13]. However, the expression and clinical pathological significance of POU2F1 in HCC are still unclear.

In this study, we reported that expression of POU2F1 was increased in HCC tissues compared with adjacent non-cancerous liver specimens, and this alteration in POU2F1 was also observed in HCC patients with metastasis compared to non- metastasis. POU2F1 over-expression was associated with a worse outcome of patients with HCC. Up-regulation of POU2F1 promoted HCC cell growth, colony formation, EMT, migration and invasion, while silencing of POU2F1 inhibited these effects. POU2F1 is a key down-stream effector of AKT pathway in the regulation of HCC malignant phenotypes. POU2F1 induced the transcription of *Twist1*, *Snai1*, *Snai2* and *ZEB1* genes which induce cancer cell EMT. This study indicates the functional roles of POU2F1 in the development and progression of HCC.

## RESULTS

### POU2F1 over-expression in HCC correlates with clinicopathological features

**Figure 1:**
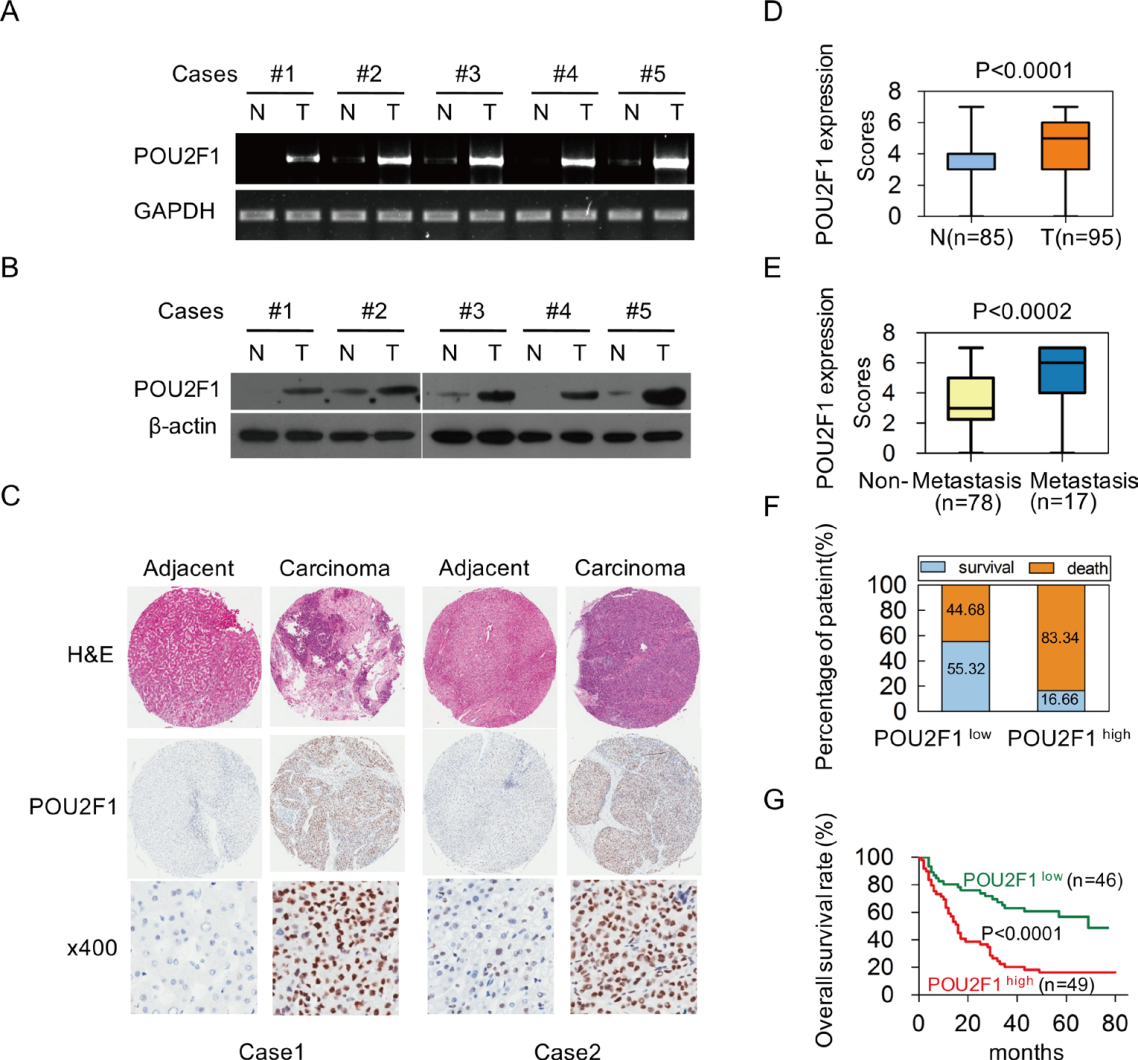
Over-expression of POU2F1 is associated with poor prognoses in patients with HCC **(A, B)** POU2F1 protein levels in five pairs of matched primary HCC (T) and corresponding adjacent non-cancerous liver tissue (N) samples were detected by RT-PCR and western blotting. **(C)** Representative IHC images of POU2F1 expression in HCC tissues and corresponding adjacent non-cancerous liver tissues. **(D)** Differences in POU2F1 expression scores between HCC tissues (T) (n = 95) and adjacent non-cancerous liver tissues (N) (n = 85) are shown as a box plot. **(E)** POU2F1 expression scores in HCC tissues of patients with metastasis (n = 17) compared those without metastasis (n = 78). **(F)** The patients with POU2F1^high^ were at a higher risk of hepatic cancer-related death than those with POU2F1^low^. **(G)** The Kaplan-Meier survival analysis shows that patients with high expression of POU2F1 had shorter OS.

To examine the role of POU2F1 in hepatic carcinogenesis, POU2F1 levels in five pairs of matched primary HCC (T) and corresponding adjacent non-tumor hepatic (N) tissue samples were analyzed. POU2F1 mRNA and protein levels were elevated in all HCC compared with matched adjacent non-tumor hepatic tissues (Figure 1A and 1B). In addition, an extensive tissue microarray analysis of 85 pairs of matched HCC and non-tumor tissues plus 10 unpaired HCC samples was performed by using an IHC assay (Figure 1C). We found that POU2F1 protein was over-expressed in 51.57% (49/95) of the HCCs, but only 25.88% (22 of 85) of the non-HCC tissues were stained positively for POU2F1, and the difference in POU2F1 staining between the HCC and adjacent non-tumor hepatic (N) tissues was statistically significant (*P* < 0.0001) (Figure 1D). Similarly, such alterations in POU2F1 were also observed in HCC tissues of patients with metastasis compared to non- metastasis (*P* < 0.0002) (Figure 1E). To find out the relevance of POU2F1 over-expression to clinicopathological features of HCC, we performed the Chi-square test. Our findings indicated that up-regulation of POU2F1 positively correlated with tumor size (≥ 5 or < 5 cm) (*P* = 0.007), histological grade (*P* = 0.019), HCC metastasis (*P* = 0.044) and the clinical stage (*P* = 0.0004) (Table 1).

**Table 1:** Correlation between clinical features and POU2F1 expression in HCC

Clinical character	Clinicalgroups	All cases	POU2F1		x^2^	*p* value
			High (%)	Low (%)		
Age (years)	≤ 60	70	38 (54.3)	32 (45.7)	1.139	0.286
	> 60	24	10 (41.7)	14 (58.3)		
Gender	Female	10	5 (50)	5 (50)	0.011	1.000*
	Male	85	44 (51.7)	41 (48.3)		
Tumor size (cm)	< 5	38	13 (34.2)	25 (65.8)	7.250	0.007
	≥ 5	56	35 (62.5)	21 (37.5)		
Cirrhosis	No	59	35 (59.3)	24 (40.7)	3.738	0.053
	Yes	36	14 (38.9)	22 (61.1)		
Histological grade	I-II	61	26 (42.6)	35 (57.4)	5.474	0.019
	III	34	23 (67.6)	11 (32.4)		
Metastasis	No	78	34 (43.6)	44 (56.4)	4.074	0.044
	Yes	17	12 (70.6)	5 (29.4)		
PVTT	No	91	47 (51.6)	44 (48.4)	0.004	1.000*
	Yes	4	2 (50)	2 (50)		
Clinical stage	I-II	43	13 (30.2)	30 (69.8)	12.529	4.000E-4
	III-IV	44	30 (68.2)	14 (31.8)		

POU2F1 high score: 4-7; POU2F1 low score: 0-3;

*: *p* value of Fisher's Exact Test;

PVTT: portal vein tumor thrombus;

Clinical stage: American Joint Committee on Cancer classification (AJCC).

### POU2F1 over-expression in HCC correlates with a poor prognosis

**Figure 2:**
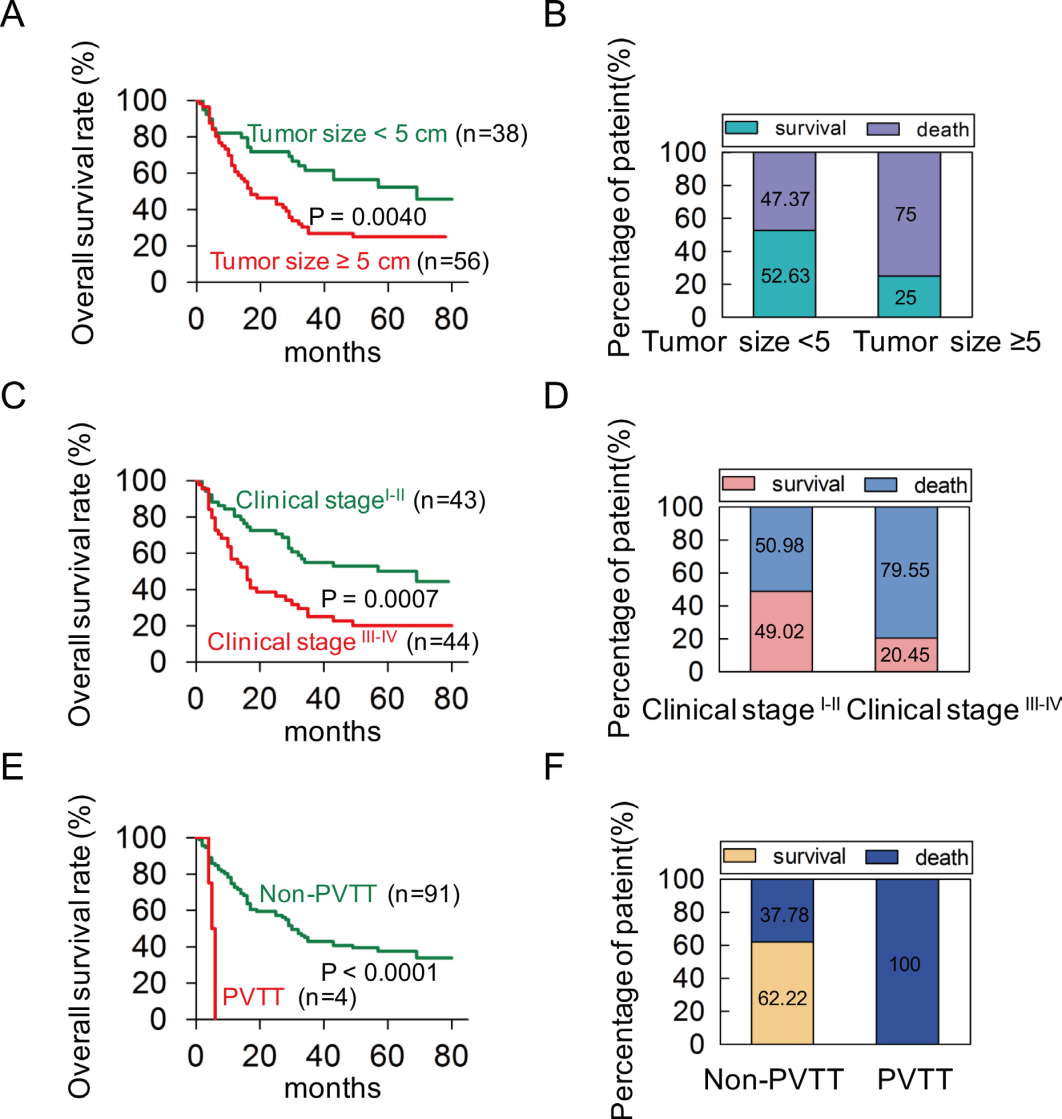
A univariate analysis was conducted to find out probable independent factors in poor prognoses of HCC patients before performing a Cox multivariate analysis **(A, B)** The patients with tumor size ≥ 5 cm had shorter OS and higher death rates in HCC. **(C, D)** The patients in clinical stage III-IV had shorter OS and higher death rates in HCC. **(E, F)** The patients with PVTT had shorter OS and higher death rates in HCC.

**Figure 3:**
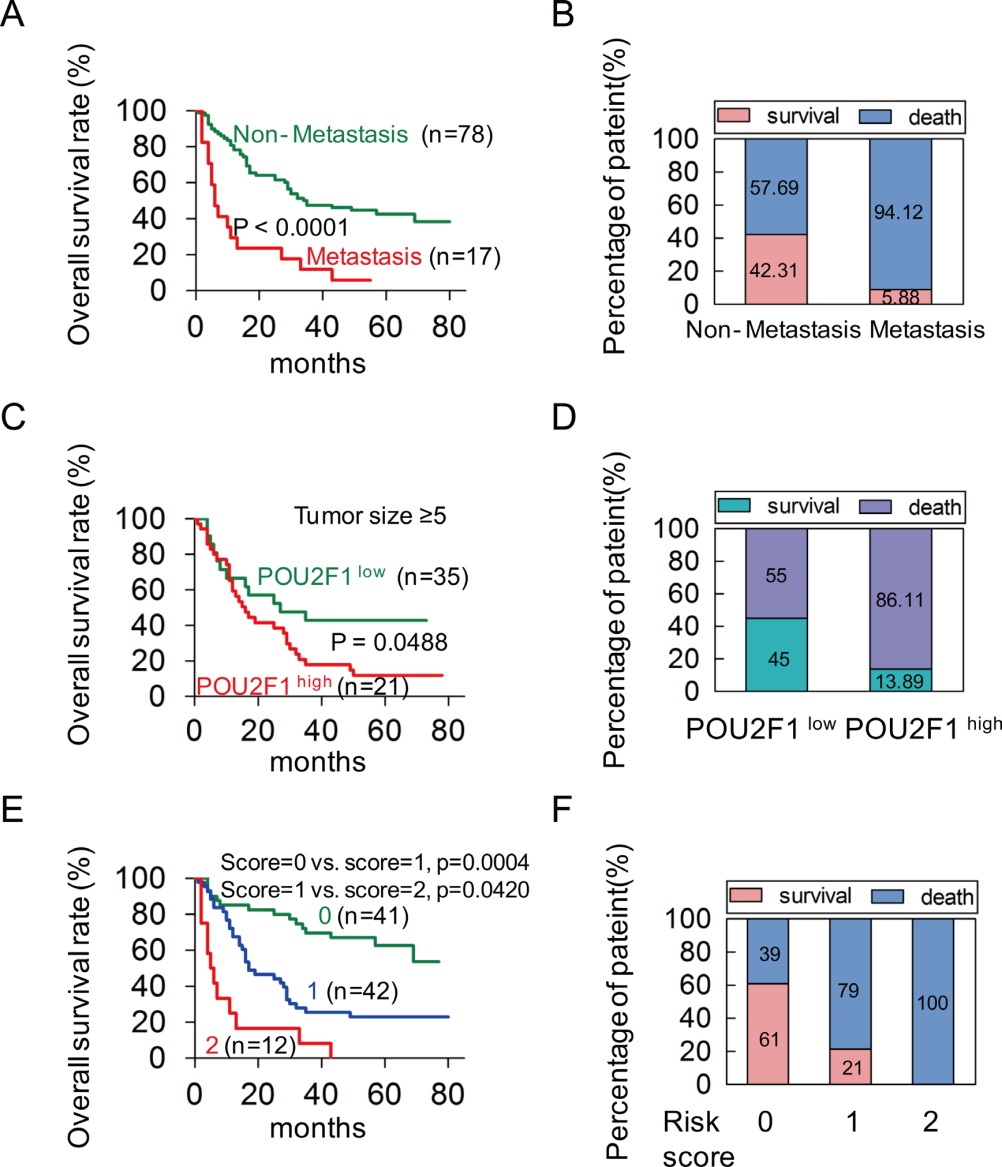
POU2F1 serves as an independent factor in a poor prognosis of HCC **(A, B)** The patients with metastasis had shorter OS and higher death rates in HCC. **(C, D)** In the tumor size ≥ 5 cm group, the patients with high expression of POU2F1 had shorter OS and higher death rates. **(E)** A Kaplan-Meier plot for the OS rates of patients with HCC in Risk score = 0, Risk score = 1 and Risk score = 2 group. **(F)** The death rates of HCC patients between the Risk score groups.

To investigate the correlation of POU2F1 levels with prognoses of HCC patients, survival analyses were conducted. Kaplan-Meier survival analyses revealed that patients with POU2F1 over-expression were at a higher risk of hepatic cancer-related death than patients with low POU2F1 expression, and they trended to have higher death rates and shorter overall survival (OS) (Figure 1F and 1G). The mean OS time for HCC patients with POU2F1 over-expression was 26.25 months, whereas that for HCC patients with low POU2F1 expression was 52.19 months (Table 2). Therefore, POU2F1 over-expression was correlated with poor prognoses of HCC patients. In addition to a high POU2F1 expression level, a tumor ≥ 5 cm, clinical stage III-IV, the presence of PVTT and HCC metastasis were also associated with shorter OS and higher death rates (Figure 2, Figure 3A, Figure 3B and Table 2). While clarifying the specific subgroups of patients that were negatively influenced by POU2F1 up-regulation, we found that POU2F1 was highly expressed in patients with tumors ≥ 5 cm, and the prognostic significance of POU2F1 was retained in this subgroup. In the subgroup of HCC patients with tumors ≥ 5 cm, high POU2F1 expression showed apparent prognostic value for predicting poorer OS and higher death rates (*P* = 0.0488) (Figure 3C and 3D). The findings elucidate that the level of POU2F1 may serve as a prognostic molecular marker for a certain subgroup of HCC patients.

**Table 2:** Association between POU2F1 expression or clinical features and OS

Clinical character	Category	All cases	OS (months)		*p* value
			Mean	95% CI	
POU2F1	Low	46	52.19	43.58–60.80	1.729E-5
	High	49	26.25	18.93–33.56	
Age (years)	≤ 60	70	35.73	28.51–42.96	0.146
	> 60	24	48.31	36.32–60.30	
Gender	Female	10	44.70	26.64–62.76	0.381
	Male	85	38.50	31.78–45.21	
Tumor size (cm)	< 5	38	51.20	41.28–61.12	0.004
	≥ 5	56	31.23	23.71–38.76	
Cirrhosis	No	59	39.24	31.19–47.29	0.885
	Yes	36	39.55	29.39–49.72	
Histological grade	I-II	61	41.82	33.92–49.73	0.246
	III	34	34.71	24.29–45.19	
Metastasis	No	78	44.85	37.86–51.84	2.377E-6
	Yes	17	13.82	6.49–21.16	
PVTT	No	91	41.05	34.54–47.56	4.600E-5
	Yes	4	5.25	4.31–6.19	
Clinical stage	I-II	43	49.13	40.64–57.63	1.078E-4
	III-IV	44	28.19	19.82–36.57	

OS: overall survival;

CI: confidence interval;

PVTT: portal vein tumor thrombus;

Clinical stage: American Joint Committee on Cancer classification (AJCC).

### POU2F1 serves as an independent prognosis factor for HCC patients

The Univariate analyses revealed that POU2F1 expression level, tumor size, PVTT, metastasis and clinical stage were significant predictors of OS (Table 3). After adjusting for other confounding factors, the two independent factors (high POU2F1 and the presence of metastasis) were used in a Forward (conditional) Cox multivariate proportional hazard model for the OS, and the hazard ratio (HR), 95% confidence interval (CI), and *P* values of these two independent predictors are listed in Table 3. The analysis revealed that high POU2F1 expression in HCC tissues and the presence of metastasis were independent predictors of the OS (Table 3). Furthermore, we established a post-operation prognostic score model by calculating the number of independent predictors (POU2F1 and metastasis) for each patient. The analysis results showed that patients with higher risk scores (RSs) showed shorter OS and higher death rates (Figure 3E and 3F). It demonstrates that the post-operation prognostic score model may be able to predict the prognosis of HCC patients.

**Table 3:** Cox multivariate proportional hazard model of independent predictors on OS

Clinical character	OS (months)			
	Univariate analysis		Multivariate analysis	
	HR (95% CI)	*p* value	HR (95% CI)	*p* value
Age (> 60 y vs. ≤ 60 y)	0.98(0.96–1.01)	0.214		
Gender (male vs. female)	1.49(0.60–3.73)	0.390		
Tumor size (≥ 5 vs. < 5, cm)	2.26(1.29–3.97)	0.004		
Cirrhosis (yes vs. no)	0.90(0.57–1.62)	0.887		
Histological grade (III vs. I-II)	1.47(0.87–2.48)	0.150		
PVTT (present vs. absent)	7.04(2.31–21.48)	0.001		
Clinical stage (III-IV vs. I-II)	2.91(1.64–5.15)	2.000E-4		
Metastasis (yes vs. no)	3.69(2.05–6.63)	1.314E-5	3.35(1.71–6.56)	4.374E-4
POU2F1 (high vs. low)	3.07(1.78–5.29)	5.141E-5	2.73(1.54–4.87)	0.001

### POU2F1 promotes HCC cell malignant phenotypes

**Figure 4:**
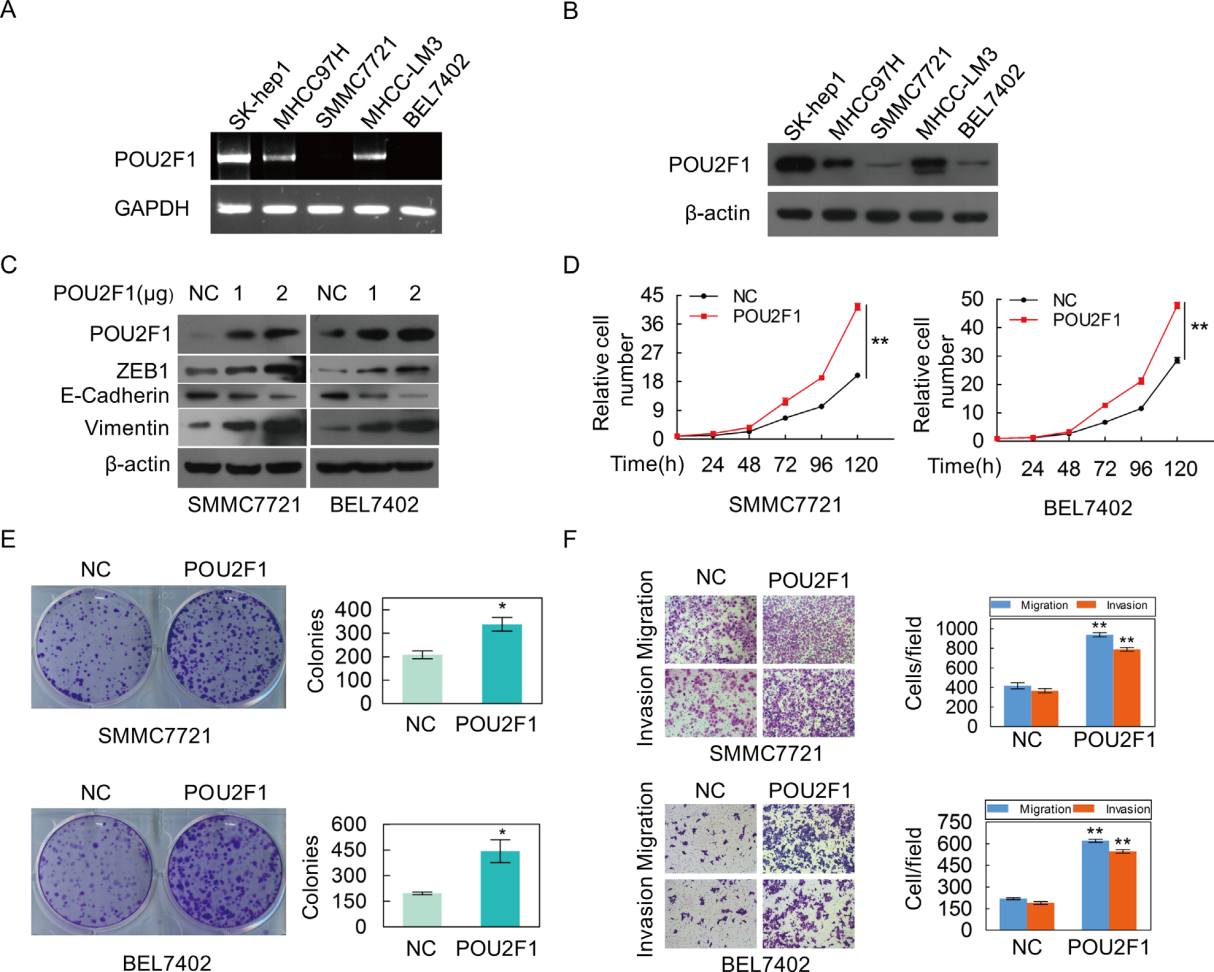
The over-expression of POU2F1 promotes the malignant phenotypes of HCC cells **(A, B)** POU2F1 protein levels in five cell lines of HCC were detected by RT-PCR and western blotting. **(C)** POU2F1 over-expression and EMT markers were detected by western blotting in SMMC7721 and BEL7402 cells. **(D-F)** The effects of POU2F1 over-expression on cell growth **(D)**, colony formation **(E)** and migration and invasion **(F)** were detected.

POU2F1 was highly expressed in three HCC cell lines (SK-hep1, MHCC-LM3 and MHCC97H), whereas low-expressed in SMMC7721 and BEL7402 cell lines (Figure 4A and 4B). To investigate the role of POU2F1 in HCC progression, POU2F1 was over-expressed in BEL7402 and SMMC7721 cells with low POU2F1. We found that over-expression of POU2F1 stimulated cell growth (Figure 4D), colony formation (Figure 4E), migration and invasion (Figure 4F) in BEL7402 and SMMC7721 cells. EMT is a crucial step during tumor cell migration and invasion; ZEB1, E-cadherin and Vimentin have been widely used as classic EMT markers [14, 15]. Here, we demonstrated that POU2F1 augmentation promoted HCC cell EMT (Figure 4C).

**Figure 5:**
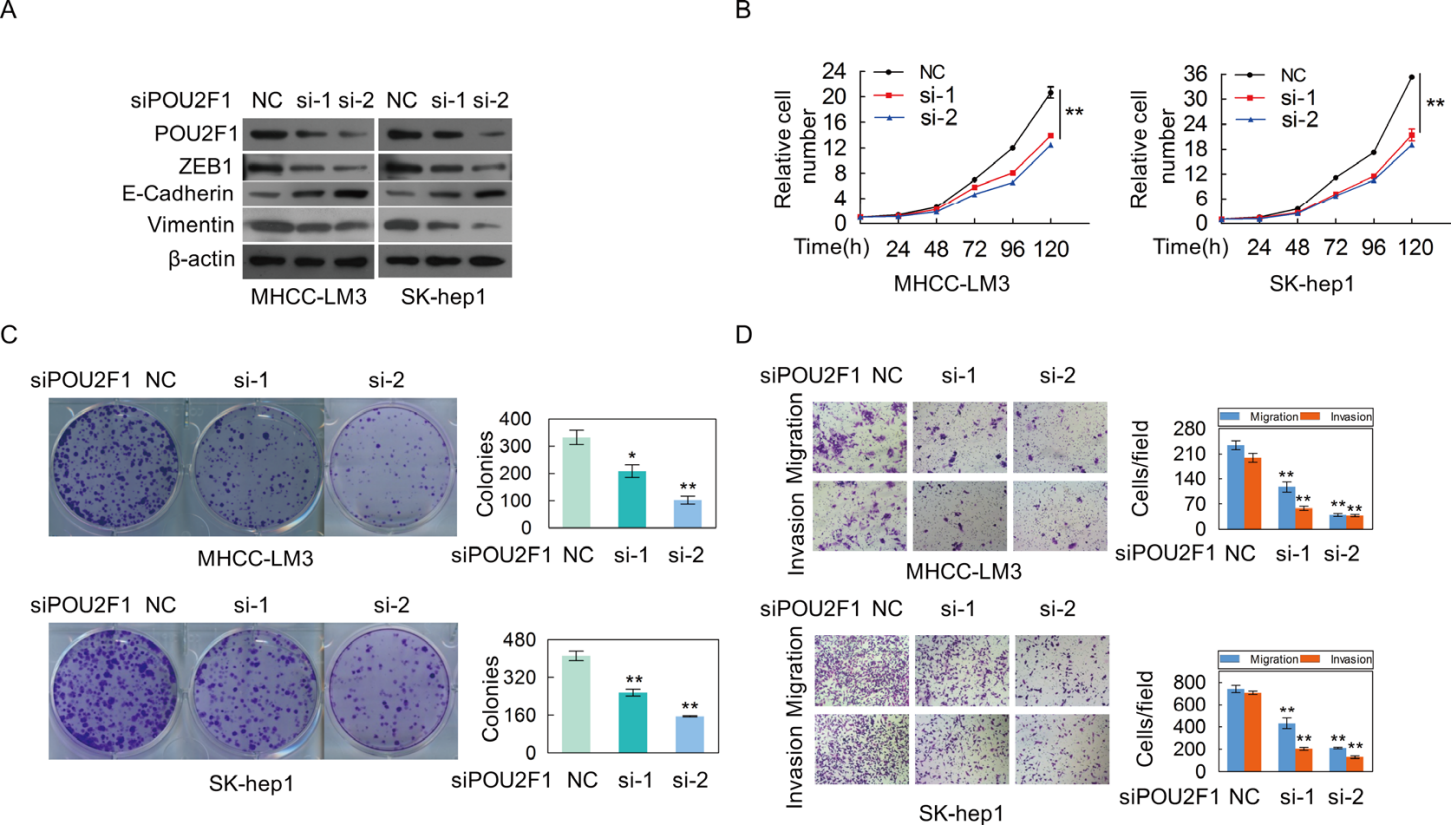
The silencing of POU2F1 inhibites the malignant phenotypes of HCC cells **(A)** POU2F1 silencing and EMT markers were detected by western blotting in MHCC-LM3 and SK-hep1. **(B-D)** The effects of POU2F1 silencing on cell growth **(B)**, colony formation **(C)** and migration and invasion **(D)** were detected.

In order to further verify its role in HCC, POU2F1 was silenced in SK-hep1 and MHCC-LM3 cells with high POU2F1. We found that silencing of POU2F1 inhibited HCC cell EMT (Figure 5A), cell growth (Figure 5B), colony formation (Figure 5C), migration and invasion (Figure 5D). Taken together, we demonstrate that POU2F1 promotes HCC cell malignant phenotypes, which matches the poor prognosis in HCC patients.

### POU2F1 is a key effector of AKT pathway on HCC cell malignant phenotypes

**Figure 6:**
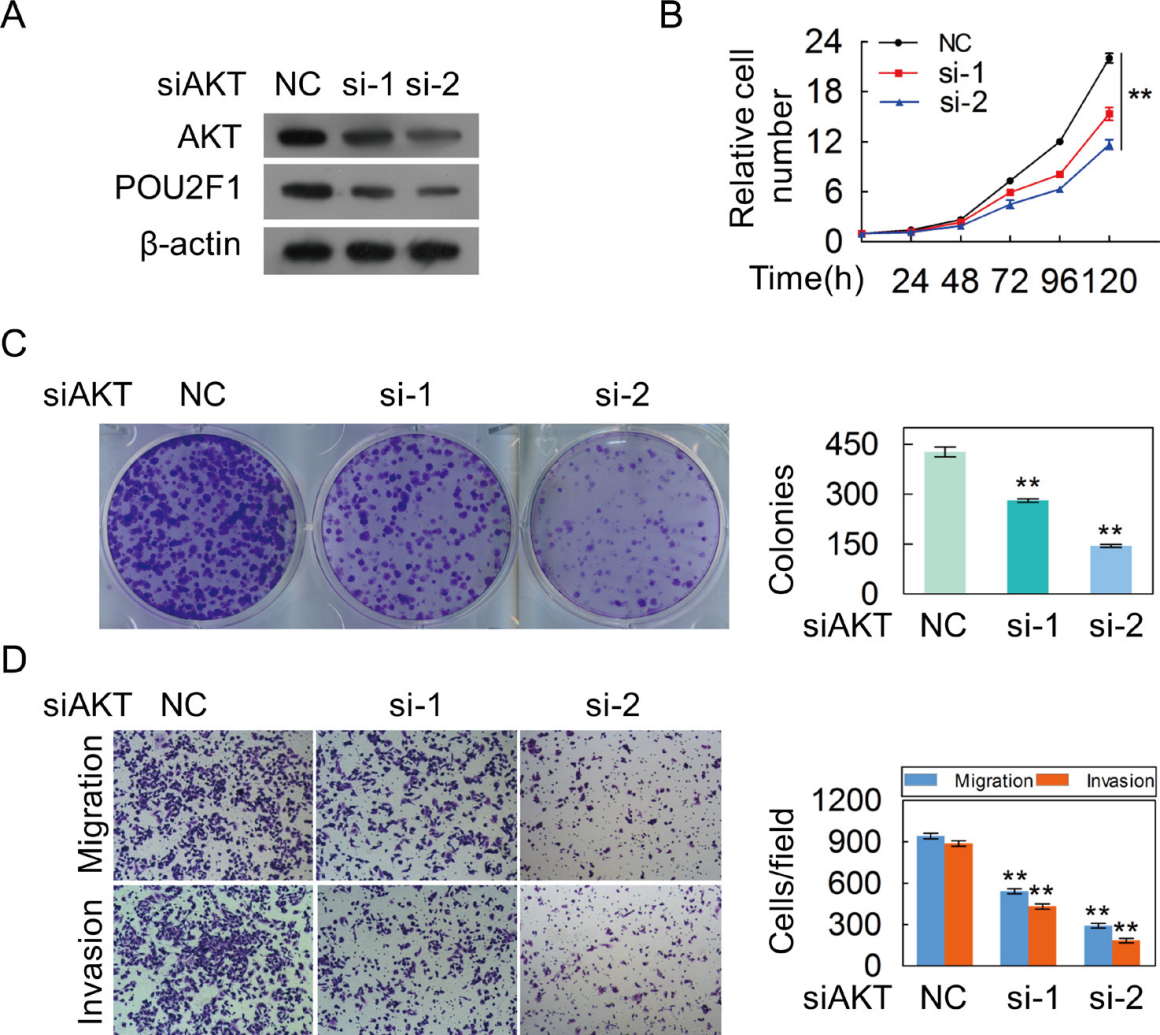
The silencing of AKT inhibites the malignant phenotypes of HCC cells **(A)** AKT and POU2F1 protein levels were detected by western blotting when AKT expression was silenced by anti-AKT siRNAs in SK-hep1 cells. **(B-D)** The effects of AKT silencing on cell growth **(B)**, colony formation **(C)** and migration and invasion **(D)** were detected.

**Figure 7:**
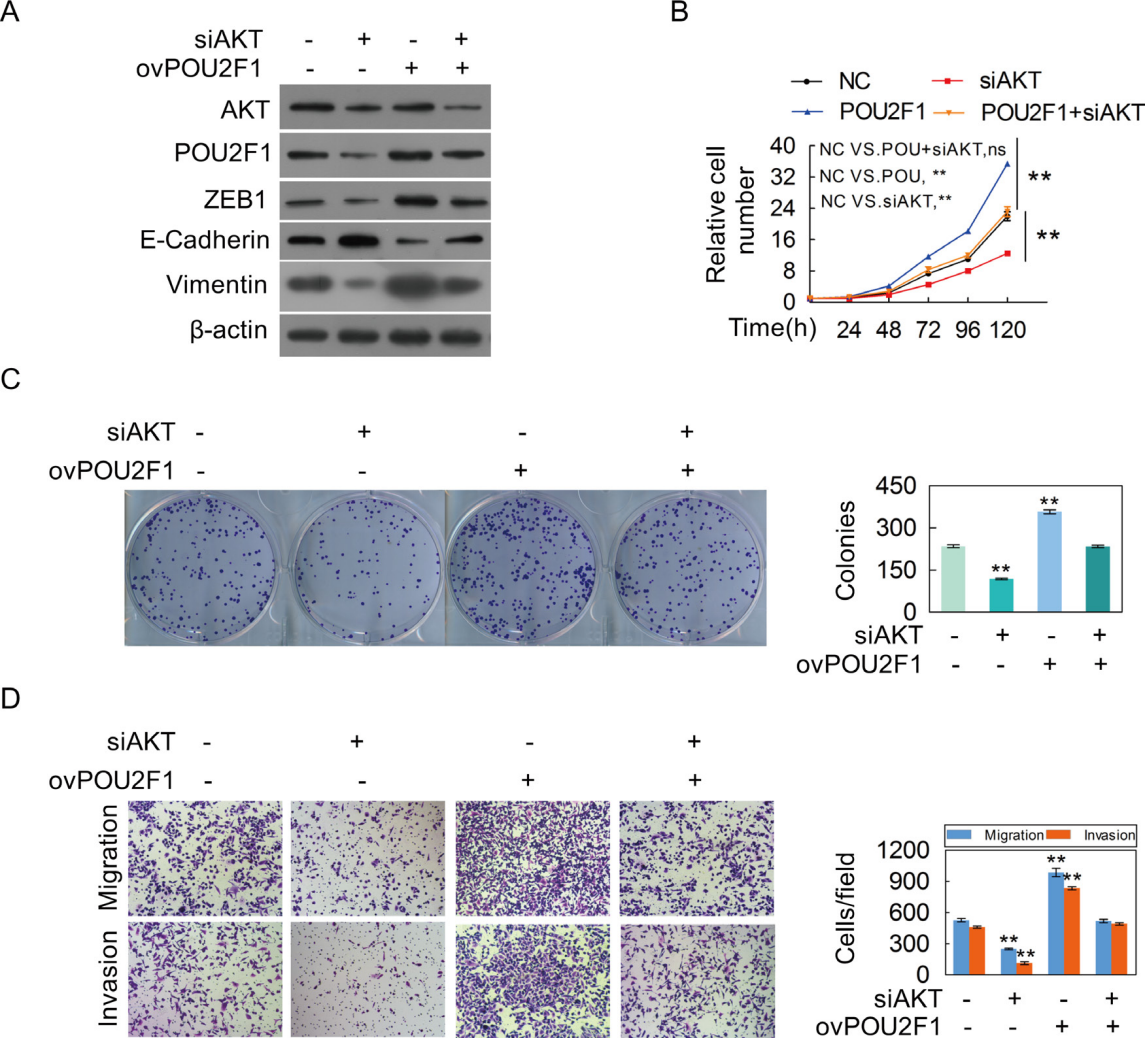
POU2F1 is a critical effector for AKT-stimulated malignant phenotypes of HCC cells **(A-D)** SK-hep1 cells were co-transfected with the POU2F1 plasmid and anti-AKT siRNA, and AKT, POU2F1 and EMT marker protein levels **(A)**, cell growth **(B)**, colony formation **(C)**, migration and invasion abilities **(D)** were determined.

We further sought to determine the molecular mechanism of POU2F1 in HCC cells. Our results showed that AKT silencing significantly decreased the amount of POU2F1 (Figure 6A). As expected, decreasing of AKT hindered malignant phenotypes of HCC cells (Figure 6B, 6C and 6D). POU2F1 over-expression could reverse the inhibitory effects of AKT knockdown on EMT (Figure 7A), cell growth (Figure 7B), colony formation (Figure 7C), migration and invasion (Figure 7D). Our results indicated that POU2F1 is a key effector of AKT pathway on HCC cell malignant phenotypes.

### POU2F1 induces the expression of EMT genes

**Figure 8:**
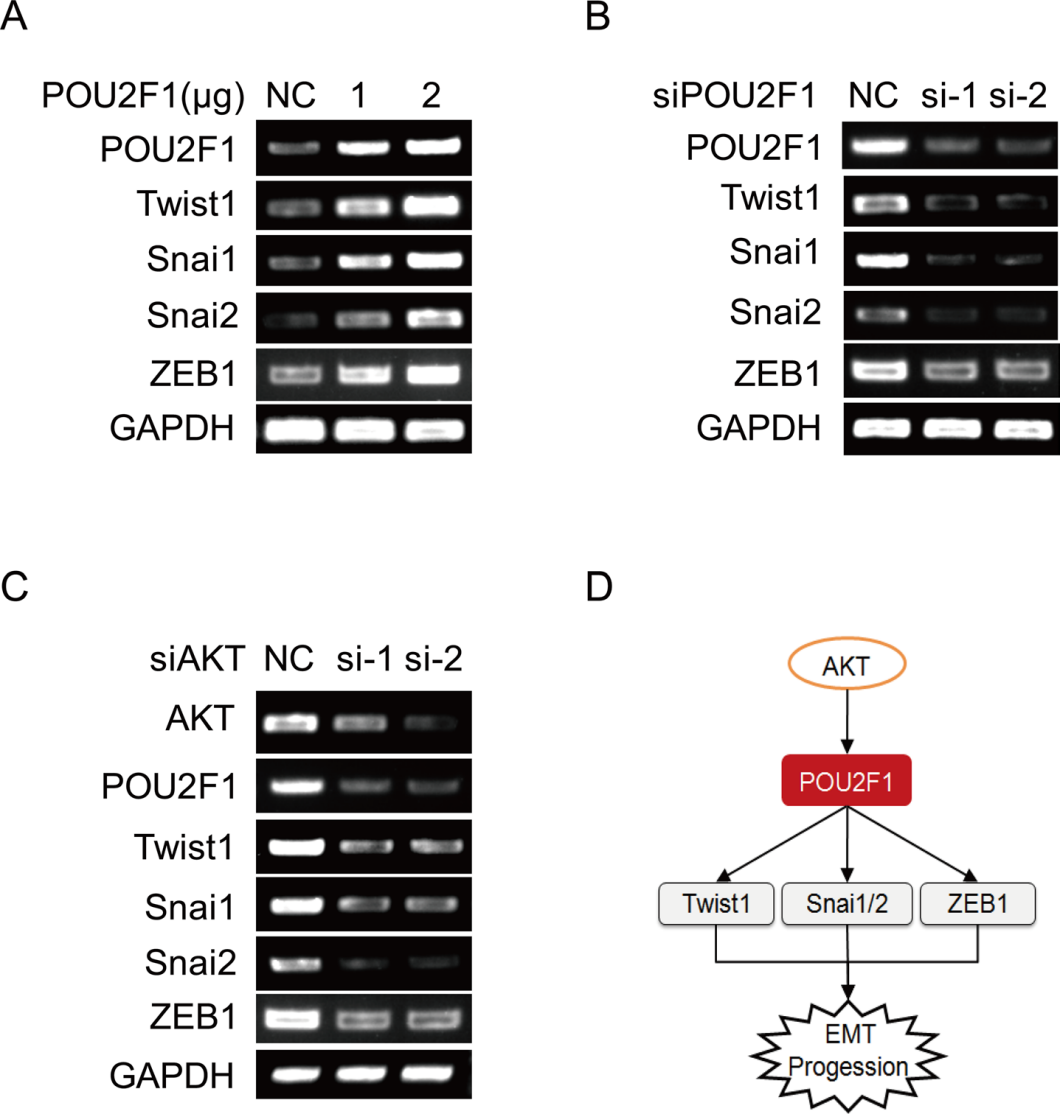
POU2F1 promotes HCC cell EMT by up-regulating the expression of EMT genes **(A)** EMT genes were detected by RT-PCR when POU2F1 was over-expressed in SMMC7721. **(B)** EMT genes were detected by RT-PCR when POU2F1 was silenced in SK-hep1. **(C)** EMT genes were detected by RT-PCR when AKT was silenced in SK-hep1. **(D)** A model of POU2F1 signaling pathway involved in HCC cell EMT.

A recent study showed that in *POU2F1* regulates the expression of *Twist1*, *Snai1* and *Snai2*, the key transcription factors that are involved in the regulation of E-cadherin, Vimentin, and EMT [16]. Here, we demonstrated that up-regulation of *POU2F1* increased mRNA levels of EMT genes such as *Twist1*, *Snai1*, *Snai2* and *ZEB1* in HCC cells, while silencing of *POU2F1* down-regulated the mRNA levels of these genes (Figure 8A and 8B). We also further found that *AKT* knockdown depressed *POU2F1*, *Twist1*, *Snai1*, *Snai2* and *ZEB1* mRNA levels in HCC cells (Figure 8C). Collectively, these findings showed that POU2F1 induced the transcription of *Twist1*, *Snai1*, *Snai2* and *ZEB1* genes which induce cancer cell EMT.

## DISCUSSION

*POU2F1* is a member of the POU homeodomain family, and it is expressed ubiquitously [17]. Analyses of databases revealed that POU2F1 showed higher expression levels in kidney, ovary, and esophageal cancer, which was compared to normal controls, whereas expression of POU2F1 in some brain tumors, bladder cancer, and liposarcoma was reduced [18]. Another study which focused specifically on intestinal-type gastric cancer showed that 74% of the 42 gastric carcinoma samples displayed an increasing POU2F1 protein level, respectively [19]. According to our results, POU2F1 protein was over-expressed in the most of HCC tissues compared with adjacent non-cancerous liver specimens. These observations indicated that it may be possible to use expression levels of POU2F1 as a diagnostic tool to distinguish HCC tissues from non-malignant liver tissues. Furthermore, over-expression of POU2F1 in HCC significantly correlated with some of the clinicopathological features, suggesting the up-regulated expression of POU2F1 may facilitate poor prognoses of HCC patients. Importantly, we further found that the high POU2F1 level in HCC was an independent predictor of overall survival, which was the same as the other clinicopathological characteristic metastasis. These findings underscore a potentially important role of POU2F1 in the diagnosis and prognosis of HCC patients.

The POU2F1 has recently been suggested to play a crucial role in the tumourigenesis of several types of human cancer [20–23]. It has been shown that POU2F1 acts not only as an oncogene but also as a tumor suppressor gene in different cancers [24–29]. Accordingly, our statistical data indicated that POU2F1 over-expression in HCC significantly correlated with clinicopathological characteristics and poor prognoses. It is therefore a logical hypothesis that POU2F1 may act as an oncogene, which can play a very important role in tumorigenesis and progression of HCC. To confirm this hypothesis, a series of *in-vitro* assays, such as proliferation, colony formation, migration and invasion assays, were employed to investigate the role of POU2F1 in HCC cell growth, EMT, migration and invasion. The results showed that POU2F1 knockdown inhibited malignant phenotypes, such as proliferation, colony formation, EMT, migration and invasion. In contrast, the ectopic over-expression of POU2F1 in HCC cells substantially promoted their aggressive phenotypes. These data support our emerging view that POU2F1 may function as an oncogene in HCC, expanding upon its previously reported role as a tumor suppressor gene in other cancers. However, by what kinds of pathway POU2F1 may be regulated and how POU2F1 stimulates HCC cell EMT are still dimness and should be investigated in the next step.

A recent study showed that POU2F1 regulates expression of the EMT genes *Twist1*, *Snai1* and *Snai2* under hypoxia-dependent loss of PER2 in breast cancer [16]. Meanwhile, relevant studies have showed that many important transcriptional factors, including *Twist1*, *Snai1*, *Snai2* and *ZEB1*, are implicated in E-cadherin transcriptional suppression as well as Vimentin activation, leading to EMT [30–33]. Moreover, our previous research has found that POU2F1 is elevated in GC tissue, and is also regulated by the AKT pathway, leading to the induction of EMT [34]. However, the molecular mechanisms of POU2F1-induced EMT in HCC are still unrevealed. Firstly, we discovered that POU2F1 is a key effector of AKT in the regulation of HCC malignant phenotypes. Further, we found that *POU2F1* activated the transcription of EMT genes that contain *Twist1*, *Snai1*, *Snai2* and *ZEB1*, controlling E-cadherin down-regulation and Vimentin up-regulation. Finally, we elucidated that transcriptional factors including *POU2F1*, *Twist1*, *Snai1*, *Snai2* and *ZEB1* expression were regulated by the AKT pathway in HCC cells. Therefore, we believe that POU2F1 regulated by AKT pathway promotes HCC aggressive phenotypes by regulating the transcription of EMT genes, which leads to the occurrence and development of EMT in HCC cells.

In summary, up-regulation of POU2F1 correlates with poor prognoses in patients with HCC. A novel role for POU2F1 in HCC tumorigenesis and progression is here elucidated, in which up-regulation of POU2F1 stimulates cell growth, colony formation, EMT, migration and invasion. POU2F1 may function as an oncogene in HCC. POU2F1 is a key effector of the AKT pathway on HCC cell malignant phenotypes. *POU2F1* promoted HCC cell EMT by up-regulating the expression of *Twist1*, *Snai1*, *Snai2*, *ZEB1* in HCC cells. POU2F1 may serve as a new prognostic factor and therapeutic target for HCC.

## MATERIALS AND METHODS

### Cell culture and tissue samples

HCC cell lines, including MHCC-LM3, MHCC97H, SK-hep1, BEL7402 and SMMC7721, were from the National Infrastructure of Cell Line Resources of China and were cultured in DMEM or 1640 supplemented with 10% fetal bovine serum (FBS) plus antibiotics at 37 °C in a 5% CO_2_ atmosphere. A total of 5 paired HCC and adjacent non-tumor tissue samples were obtained from patients with HCC who underwent surgery at the Third Affiliated Hospital, Guangzhou Medical University. All patients were confirmed to have HCC via histopathological evaluations. None of the patients had been treated before surgery. Detailed information pertaining to clinicopathological characteristics was recorded. All tissue samples were rapidly snap-frozen in liquid nitrogen and stored at -196 °C until RNA and protein extraction. Informed consent was obtained from all patients. Our study was approved by the Research Ethics Committee of the Third Affiliated Hospital of Guangzhou Medical University, China. Tissue microarray chips comprising 85 pairs of matched HCC and non-cancerous liver tissue plus 10 HCC tissue samples and their clinicopathological information were purchased from Shanghai OUTDO Biotech Co, Ltd (Shanghai).

### Immunohistochemistry (IHC) staining and stratification of POU2F1 expression in HCC

IHC assays were performed on tissue microarray chips according to a standard labelled streptavidin biotin (LSAB) protocol (Dako, Carpinteria, CA, USA) with anti-POU2F1 antibodies. Nuclear staining of the biomarkers was evaluated by two independent clinical pathologists using the German semi-quantitative scoring system which is according to the staining intensity (no or slight staining, moderate staining and strong staining, were allotted scores of 1, 2 and 3, respectively) and the percentage of positive cells (≤ 10% positive cells, 11-50% positive cells, 51-80% positive cells and > 80% positive cells were allotted scores of 0, 2, 3 and 4, respectively), as previously described [35]. POU2F1 expression scores for the percentage of positive cells and the staining intensity were added. POU2F1 expression levels were dichotomized as low expression (scored 0-3) and high expression (scored 4-7) in HCC tissues or non-cancerous liver tissues.

### Migration and invasion assays

*In vitro* migration and invasion assays were performed using transwell chambers as previously described [36]. Briefly, cells were transfected with the indicated concentrations of the indicated siRNAs or plasmids for 48 h, respectively. Cell migration and invasion were then measured using transwell chambers with 8.0 μm pore membranes (BD, NY, USA). Cells on the undersurface were stained with 5% crystal violet and counted under a microscope.

### Colony formation assays

One thousand cells were plated in 6-well culture plates and cultured for two weeks. These cells were then fixed with methanol and stained with crystal violet solution. The numbers of colonies containing ≥ 30 cells were counted under the microscope. These experiments were repeated three times.

### Plasmid constructs and transfection

A POU2F1 expression plasmid (PCMV-POU2F1-flag) was constructed with synthetic oligonucleotides and the PCMV vector. BEL7402 and SMMC7721 cells were seeded in 6-well plates the day before transfection and infected with PCMV-POU2F1-flag (1 ug per well), PCMV-N-flag (2 ug per well) or a negative-control (NC) vector using lipofectamine 2000 (Invitrogen).

### RNA interference

SiRNAs against POU2F1 gene and corresponding scrambled siRNAs (GenePharma) were transfected into SK-hep1 or MHCC-LM3 cells with RNAiMAX (Invitrogen) for 48 h (unless otherwise stated). The following siRNA sequences were used: siPOU2F1#1, sense: 5’-GCCAAGACCUUCAAACAAATT-3’, anti-sense: 5’-UUUGUUUGAAGGUCUUGGCTT-3’; siPOU2F1#2, sense: 5’-CAGCAGCUCACCUAUUAA ATT-3’, anti-sense: 5’-UUUAAUAGGUGAGCU GCUGTT-3’; siPOU2F1#3, sense: 5’-CUGC UGCUCAGUCUUUAAATT-3’; antisense: 5’-UUUAA AGACUGAGCAGCAGTT-3’. We chose two of them, siPOU2F1#1 and siPOU2F1#2, for relatively higher interference efficiency. The negative control siRNA sequences were as previously reported [34].

### RT-PCR assays

Total RNA was isolated from cultured cells with Trizol reagent (Invitrogen). The cDNA synthesis was performed using PrimeScript RT-polymerase (Thermo). For the reverse transcription polymerase chain reaction (RT-PCR), the upstream primer sequence for POU2F1 was 5’-GCGAAGCTTGTTAAAATATTCAAAATGGCGGAC-3’, and the downstream primer sequence was 5’-GACTCTAGACAATCACACTGCAGAGTGAAAAAG-3’. After an initial denaturation carried out at 95 °C for 3 min, amplification was carried out by 25 cycles of denaturation at 95°C for 30 s, annealing at 58 °C for 1 min, extension at 72 °C for 30 s, and a final incubation was at 72 °C for 7 min. GAPDH was used as an internal reference for calculating relative expression levels. The upstream primer sequence for GAPDH was 5’-CGGAGTCAACGGATTTGGTCGTAT-3’, and the downstream primer sequence was 5’-AGCCTTCTCCATGGTGGTGAAGAC-3’.

### Western blotting

In brief, protein extracts were separated by 10% SDS-PAGE and then electroblotted onto PVDF membranes, which were then incubated with the indicated primary antibodies at 4 °C overnight, followed by incubation with the corresponding secondary antibodies at room temperature. The primary antibodies were as follows: POU2F1 (10387-1-AP, Proteintech, IL, USA; and β-actin (sc-81178, Santa Cruz, TX, USA).

### Statistical analysis

The SPSS 16.0 software program was used for the statistical analyses. The Pearson Chi-square test was used to compare qualitative variables, and quantitative variables were analyzed by the independent t-test. The survival probability was estimated by the Kaplan-Meier method, and the log-rank test was used to compare the survival curves between the groups. The Forward (conditional) Cox proportional hazard model was used to identify independent predictors associated with the OS. Differences between the mean values were considered significant when *P* < 0.05. In the post-operation prognostic score model that we established, each factor was allotted a score of 1, and then patients were divided into four categories by their risk scores (RSs) (0, 1, and 2). For example, “RS = 0” means patients without any of the above factors; “RS = 2” means patients with all two factors.
